# Exploring Anisotropy Contributions in Mn_*x*_Co_1–*x*_Fe_2_O_4_ Ferrite Nanoparticles for Biomedical Applications

**DOI:** 10.1021/acsanm.4c05231

**Published:** 2024-11-15

**Authors:** Marianna Gerina, Marco Sanna Angotzi, Valentina Mameli, Michal Mazur, Nicoletta Rusta, Elena Balica, Pavol Hrubovcak, Carla Cannas, Dirk Honecker, Dominika Zákutná

**Affiliations:** † Department of Inorganic Chemistry, Charles University, Hlavova 2030/8, 128 43 Prague 2, Czech Republic; ‡ Department of Chemical and Geological Sciences, University of Cagliari, S.S. 554 Bivio per Sestu, 09042 8 Monserrato, CA, Italy; § Department of Physical and Macromolecular Chemistry, Charles University, Hlavova 2030/8, 128 43 Prague 2, Czech Republic; ∥ Dipartimento di Chimica and INSTM, Universita‘di Firenze, Via della Lastruccia 3, I-50019 Sesto Fiorentino, Italy; ⊥ Institute of Physics, Faculty of Science, P.J. Šafárik University, Park Angelinum 9, 04001 Košice, Slovakia; # ISIS Neutron and Muon Facility, Science and Technology Facilities Council, Rutherford Appleton Laboratory, OX11 0QX Didcot, U.K.

**Keywords:** surface anisotropy, shape anisotropy, dipolar interactions, inhomogeneous spin structure, magnetic small-angle neutron scattering, magnetocrystalline anisotropy

## Abstract

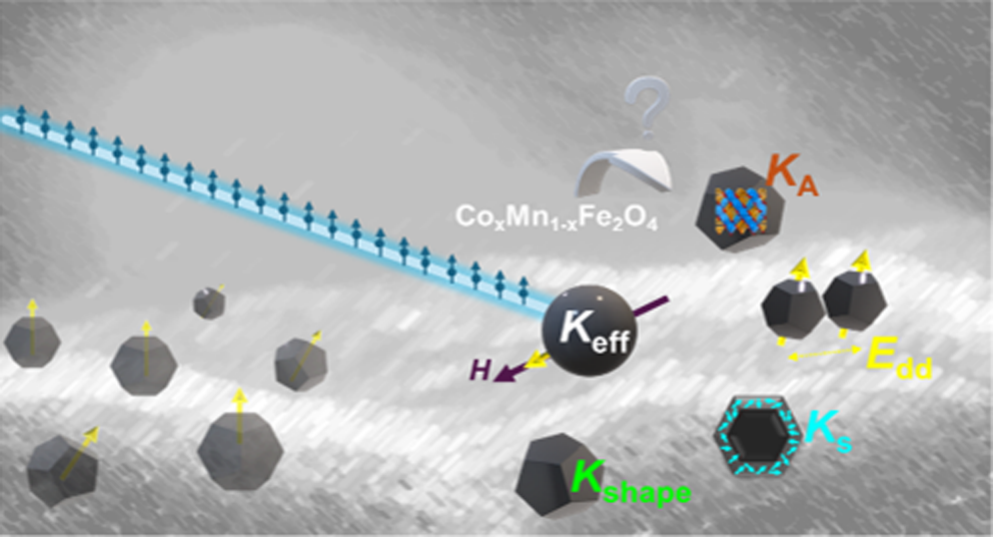

Designing well-defined magnetic nanomaterials is crucial for various applications, and it demands a comprehensive understanding of their magnetic properties at the microscopic level. In this study, we investigate the contributions to the total anisotropy of Mn/Co mixed spinel nanoparticles. By employing neutron measurements sensitive to the spatially resolved surface anisotropy with sub-Å space resolution, we reveal an additional contribution to the anisotropy constant arising from shape anisotropy and interparticle interactions. Our findings shed light on the intricate interplay among chemical composition, microstructure, morphology, and surface effects, providing valuable insights for the design of advanced magnetic nanomaterials for AC biomedical applications, such as cancer treatment by magnetic fluid hyperthermia.

## Introduction

**Figure 1:**
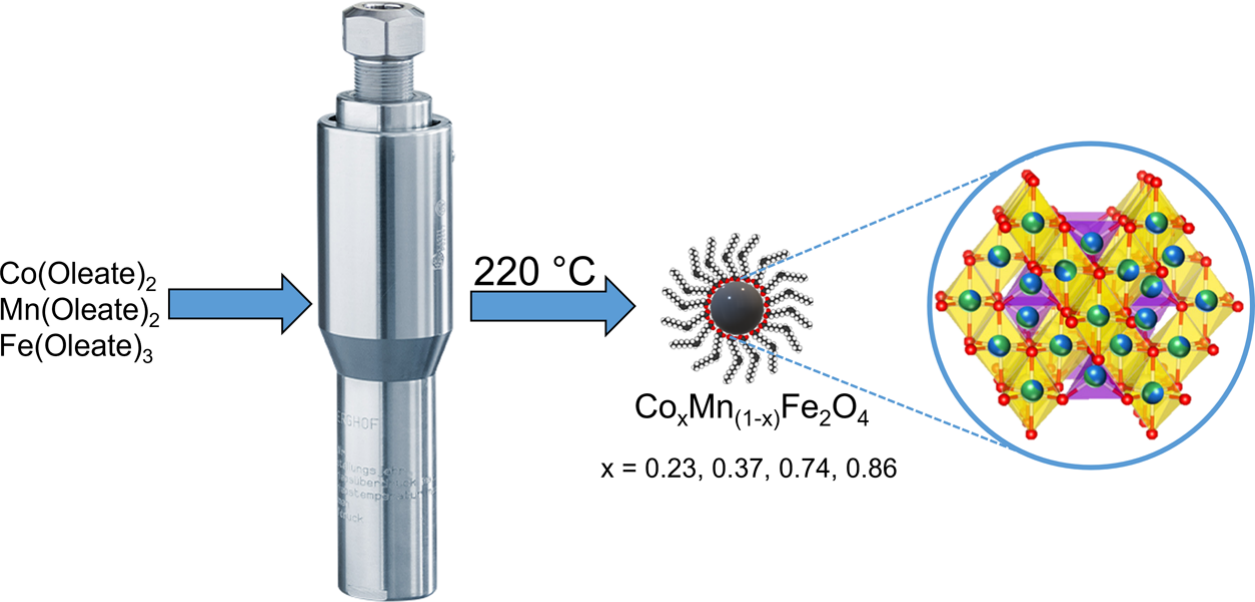
Scheme of the solvothermal synthesis of Co_*x*_Mn_1–*x*_Fe_2_O_4_.

The magnetic properties of spinel ferrite nanoparticles (NPs) result from a multifaceted interaction of factors such as chemical composition, microstructure, morphology, and surface phenomena.^1^ Among the spinel ferrites, cobalt ferrite stands out by exhibiting magnetically hard behavior, which is attributed to the high single-ion anisotropy of cobalt ions. Since the 2000s, strategies for fine-tuning its magnetic behavior and reducing its toxicity through cation substitution (Mn, Zn, etc.) have been suggested.^2^ However, establishing a correlation between the magnetic properties of NPs and their performance can pose a challenge due to the simultaneous occurrence of diverse structural and magnetic phenomena. A suite of techniques is typically employed to elucidate the interconnections between the structural and magnetic properties of NPs. These include transmission electron microscopy (TEM), dynamic light scattering (DLS), small-angle X-ray scattering (SAXS), DC magnetization, and AC susceptibility (ACS) measurements.^3^ The theoretical framework outlines the relationships between particle structure and magnetic properties in the ideal scenario of homogeneously magnetized, single-domain, and noninteracting particles. However, recent findings underscore that NP systems are more intricate than previously assumed, even for highly crystalline, monodisperse, and noninteracting NP ensembles. Indeed, several factors can affect the material’s magnetic properties, such as spin disorder and dipolar interactions. Spin disorder, i.e., random orientation of magnetic spins, is an inherent characteristic of magnetic NPs, notably when their diameter is reduced to a few nanometers.^4^ The phenomena of spin canting (defined as a partial alignment of the spins at the surface under high magnetic fields, i.e., noncollinear magnetic spin structures) and spin frustration (presence of surface atoms with a reduced number of magnetic neighbor ions around) are frequently analyzed in the context of surface effects, attributed to the unsaturated covalent bonds of surface atoms. This perspective presumes that the entire NP is crystallographically perfect, with only the surface atoms^4^ exhibiting distinct magnetic properties due to symmetry disruption. However, in actual samples, the atomic structural arrangement near the surface is considerably more intricate, with a multitude of lattice-level perturbations such as point and line defects, local strains, variations in the degree of inversion in the spinel structure, and cation depletion, to name a few. These factors may result in disordered magnetic phases or a disordered shell of a few nanometers thicknesses,^4,5^ the extent of which is dependent on the chemical composition and synthesis method.^6^ For instance, in the case of highly crystalline cobalt ferrite NPs devoid of internal defects, the spin disorder permeates the entire particle, resulting in the formation of a uniformly disordered phase.^7^ These observations imply that the spin disorder at the surface triggers a comprehensive spin reorganization within the particle. The presence of cobalt tends to favor the extended disordered configuration, particularly for larger NPs. At the same time, magnetically soft ferrites are prone to form more diverse spin configurations, even though the magnetic core–shell structure is one of the prevalent ones.^8^ A type of magnetic core–shell structure, primarily originating from the structural disorder, has been reported for cobalt ferrite NPs.^6^ Moreover, additional spin canting arises from intra- or interparticle dipolar interactions,^9^ known, for instance, to alter the heating efficiency of NPs. These dipolar interactions, leading to particle agglomeration, introduce an element of uncertainty in the determination of the effective anisotropy constant (*K*_eff_) due to the lack of experimental control over the colloidal state of individual magnetic NPs during the measurement process.^3^ This factor could account for discrepancies between the ferrofluid results and those derived from NPs supported in solid matrices or powdered preparations.^10^ As observed, disorder effects, being inherent to the materials themselves, play a crucial role in determining their magnetization properties, including coercivity, superparamagnetism, exchange interactions, and spontaneous magnetization.^11^ These properties are of paramount importance for the wide range of technological applications of magnetic NPs, such as in batteries,^12^ biomedicine,^13^ or catalysis.^14^ Surface spin canting or disorder in magnetic NPs can only be indirectly accessed using magnetization measurements, ferromagnetic resonance, Mössbauer spectroscopy,^15^ X-ray magnetic circular dichroism,^16^ and electron energy loss spectroscopy.^17^ There are probes that can provide essential information on the local distribution of the magnetic moment, such as electron holography^18^ and electron magnetic circular dichroism.^19^ Magnetic small-angle neutron scattering (SANS) is a versatile technique to directly obtain spatially sensitive information about the spin structure in NPs on the relevant nanometer length scale from the whole sample volume. SANS was utilized as a methodological approach to examine the interaction within ensembles of NPs,^20^ to probe the response of magnetic colloids^21^ and ferrofluids^22^ when subjected to external fields. The application of half-polarized SANS (SANSPOL) facilitates the resolution of the quantitative distribution of magnetization within the NPs. This confirmed the existence of spin disorder at the particle’s surface while concurrently uncovering a substantial degree of spin disorder pervading the entire NP.^23^ Recently, using the oleate-based solvothermal method (see Figure 1), we synthesized highly crystalline spheroidal Mn_*x*_Co_1–*x*_Fe_2_O_4_ NPs, having Co/Mn ratios of 6.1, 2.8, 0.6, and 0.3 for Co86Mn14, Co74Mn26, Co37Mn63, and Co23Mn77, respectively, and narrow size distribution (dispersity below 20%) and studied their macroscopic magnetic properties as a function of the chemical composition.^24^

**Figure 2:**
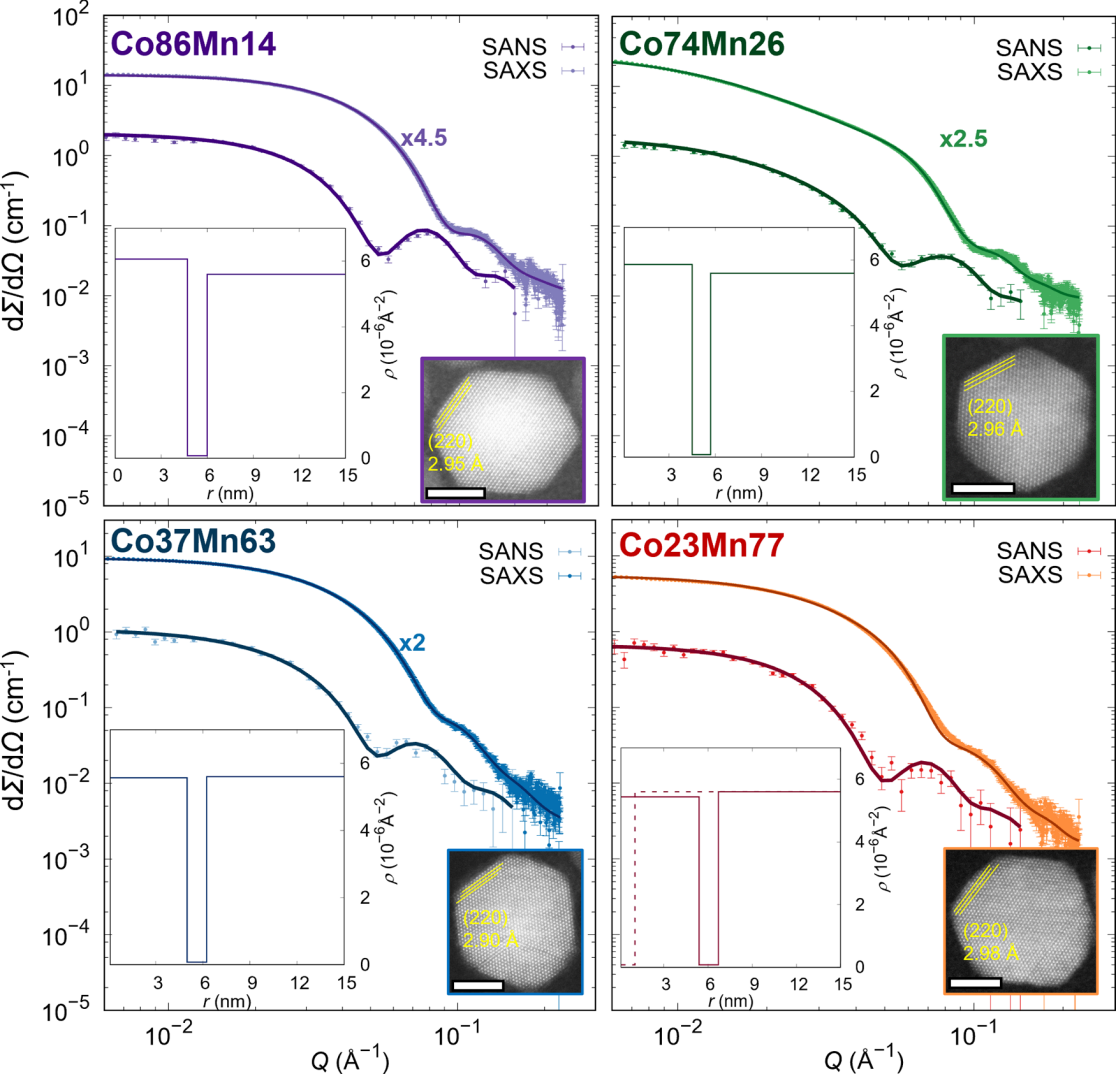
SAXS and pure nuclear SANS cross sections (points) with form factor refinements (full lines). The SAXS data were scaled by 4.5, 2.5, and 2 times, as indicated in the graphs above the SAXS data. Insets: left bottom corner—obtained radial distribution of ρ_n_ SANS refinements. Continuous lines refer to the particle, and dashed lines correspond to extra free oleic acid micelles. Right bottom corner—HRSTEM micrograph (scale bars: 5 nm).

We observed a decrease in the anisotropy (see Table S1) with increasing Mn content and its resulting influence on *T*_max_, *T*_diff_, *T*_b_, and *H*_c_ (maximum and furcation point of the ZFC curve, blocking temperature, and coercivity, respectively).^24^ All of the samples showed a deviation from the ideal case of noninteracting NPs observed in the IRM-DCD protocols as negative Δ*M* peaks in the Co-richer samples (dominant dipolar interactions) and positive Δ*M* for the Mn-richest sample (probably due to unmasked cubic anisotropy as a result of weaker dipolar interactions). Nevertheless, discerning the cause of the nonideal behavior in our samples was not possible with the macroscopic techniques used in the previous work; thus, in this work, we further investigated them through SANSPOL. The measurements were conducted on the colloidal dispersions to limit the role of interparticle interactions. Therefore, the surface anisotropy constant and the magnetocrystalline anisotropy were obtained and correlated with the Mn content. The combined refinements of the SAXS and nuclear SANS scattering cross sections (Figure 2) revealed the spheroidal morphology of Mn-mixed cobalt ferrite NPs. For both X-rays and neutrons, the scattering length densities of the inorganic cores were calculated from structural information obtained from Rietveld refinement and elemental content from the ICP experiments, previously presented in the paper of Angotzi et al.^24^ The scattering length densities of the cores, oleic acid (OA), and solvent (toluene and *d*_8_-toluene for X-rays and neutrons, respectively) were kept fixed during the refinement of the nuclear structure, and only the size of the inorganic particle, polydispersity, and length of OA were refined. The SAXS data were fitted using the spherical form factor, and at first glance, except for the Co74Mn26 sample, they show the presence of the intensity plateau known as the Guinier plateau,^25^ representing a noninteracting NP system. In the Co74Mn26 sample, the non-negligible interparticle interactions are visible as an increase in the scattering intensity in the *Q*-range of 0.02–0.05 Å^–1^. Nevertheless, after detailed analysis, only the sample Co86Mn14 did not show interparticle interactions. On the remaining samples, the structure factor “sticky-hard sphere” was included, suggesting the presence of non-negligible, although weak, particle interactions. Thus, the samples were centrifuged at 6500 rpm to separate the NPs agglomerates before SANS analysis. The SANS data were best described with the core–shell form factor, where the core corresponds to the inorganic NP size, and the shell corresponds to the oleic acid (OA) molecule at the NP surface. In the case of the Co23Mn77 sample, the nuclear SANS scattering cross section revealed a large background contribution arising from the presence of free OA micelles in the dispersion, and thus, their form factor was included in the refinement (their nuclear scattering length density is depicted as a dashed line). In all samples, the obtained thickness of OA surfactant was in the range of 1.2–1.3 nm, which agrees with previous studies.^23^ All samples have a size distribution below 20% (Table 1), representing minimal size effects on the NPs’ magnetic response. The resolved nuclear morphology of Mn-mixed cobalt ferrite nanospheres was then fixed for the description of the SANS cross section with various incident beam polarizations (*I*_Q_^–^, *I*_Q_^+^).

**Table 1:** Summarized Results from SAXS and Nuclear SANS Refinements, where *d*_nuc_, *d*_OA_, ρ_n_, and σ Correspond to the Nuclear Particle Diameter, the Thickness of the Capping Agent, Nuclear Scattering Length Density of the Core, and Log-Normal Size Distribution, Respectively

				parameter	
sample	composition	*d*_nuc_ (nm)	*d*_OA_ (nm)	ρ_n_ (10^–6^ Å^–2^)	σ (%)
Co86Mn14	Co_0.77_Mn_0.13_Fe_2.07_O_4_	9.4(1)	1.31(1)	6.062	14.9(1)
Co74Mn26	Co_0.66_Mn_0.23_Fe_2.07_O_4_	9.0(1)	1.20(1)	5.873	18.9(1)
Co37Mn63	Co_0.31_Mn_0.52_Fe_2.11_O_4_	10.0(1)	1.25(1)	5.572	17.8(1)
Co23Mn77	Co_0.19_Mn_0.65_Fe_2.11_O_4_	10.8(1)	1.31(1)	5.439	16.7(1)

**Figure 3:**
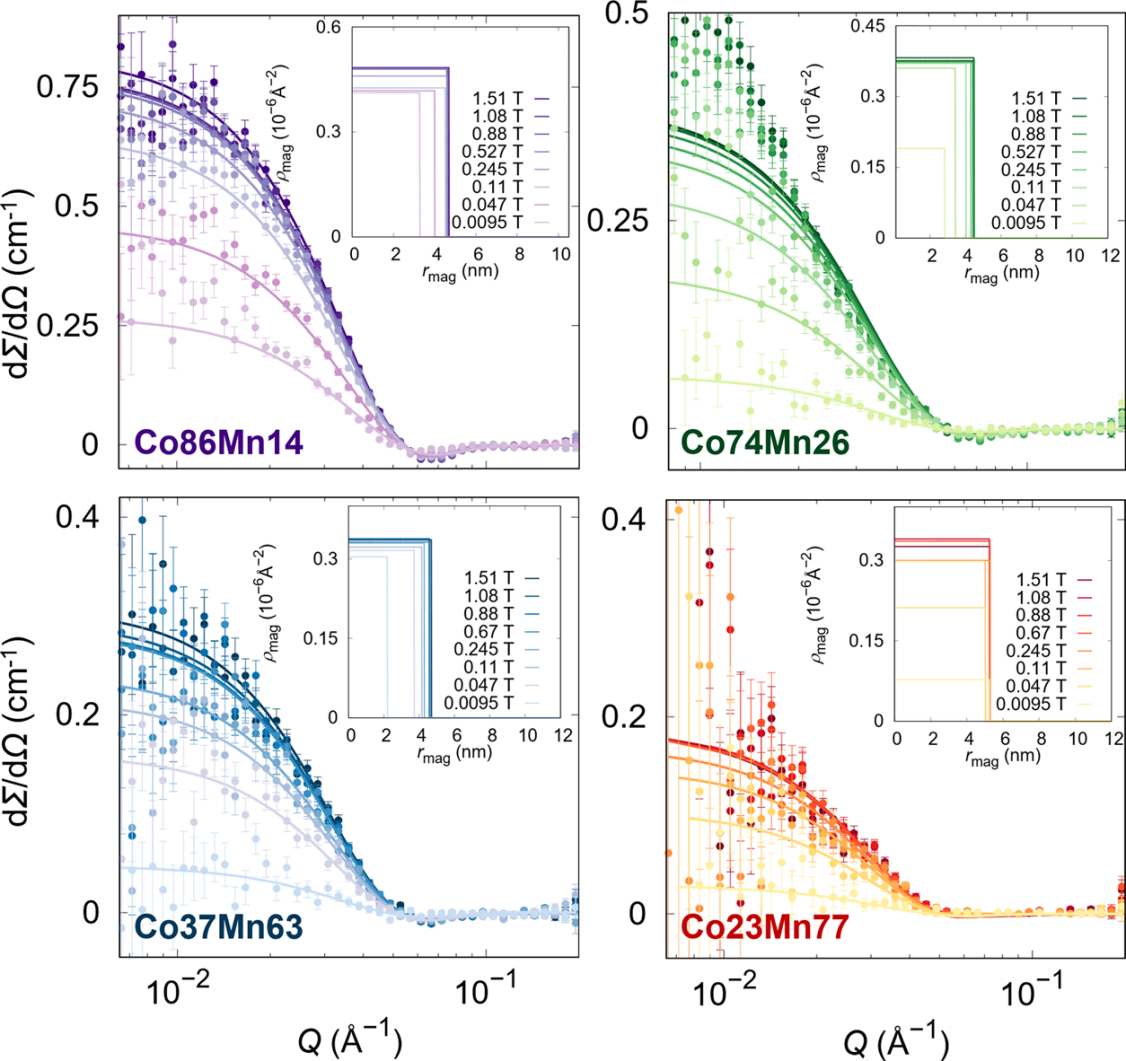
SANSPOL magnetic interference term (points) with core–shell disorder layer form factor refinements (full lines) at different applied magnetic fields. Insets: obtained radial distribution of ρ_mag_ at different applied magnetic fields.

In our previous work,^24^ the effective anisotropy constant obtained from magnetometry was found to decrease with the amount of Mn (see Table S1). To investigate more deeply the cause of the change in the effective anisotropy constant and the spin morphology of the NPs, we performed SANSPOL experiments. All samples show splitting of *I*_Q_^–^ and *I*_Q_^+^ scattering cross sections arising from the core magnetic scattering (see Figure S1 and Table S2). The data were described by the core–shell-surfactant model, where the whole physical inorganic NP size is fixed to the nuclear size obtained from SAXS and nuclear SANS, the shell represents the inorganic part with no magnetic scattering contribution that arises from spin disorder or canting, i.e., a disorder layer (*r*_nuc_ = *r*_mag_ + *d*_dis_), and the surfactant is the OA on the surface. To ensure no contribution from misaligned spins, we also performed the refinement of the nuclear-magnetic interference term (Figure 3, and the obtained results are summarized in Table S3) by subtracting *I*_Q_^–^ from *I*_Q_^+^, leading to similar observations. In fact, the samples Co89Mn14, Co74Mn26, and Co23Mn77 show the same behavior. Conversely, the sample Co37Mn63 shows the presence of a disordered layer and a change in ρ_mag_ similar to the Co-richest sample. The only refined parameters were the magnetic scattering length density of the core and the shell thickness without magnetic contribution. The radial distribution of magnetic scattering length density reported in the insets of Figure 3 shows that the Co23Mn77 sample has a negligible change in the nonmagnetized shell thickness, while the magnetized volume of the samples Co86Mn14, Co74Mn26, and Co37Mn63, richer in cobalt, is slightly increasing with an applied magnetic field. This could suggest the correlation between the presence of surface spin disorder and the amount of Co, which might also be related to a different bond strength between oleate molecules and other cations (Mn^2+^, Co^2+^, and Fe^3+^).

The longitudinal magnetization, *M*_*z*_(*H*), of all samples was accessed by refining the field dependence of magnetic SANS cross sections and nuclear-magnetic interference term. The decrease in the longitudinal magnetization with Mn content in the sample was observed, which agrees with our already published observations from macroscopic magnetization measurements.^24^ Moreover, the magnetic anisotropy was found to change throughout the series due to different cobalt contents and the interplay between anisotropy and interparticle interactions. In fact, as a result of IRM-DCD protocols, except for the sample Co23Mn77, the Δ*M* plots showed negative values of Δ*M* and an upward concave curve in the Henkels plot as an indication of demagnetizing dipolar interaction or inhomogeneous spin structure.^26^ The probability of having crystalline coherence between particles that would give rise to exchange interactions between particles was found to be very low in a previous study,^27^ favoring inhomogeneous spin structure. On the contrary, the sample Co23Mn77 showed positive values of Δ*M* and a downward concave curve, which in the literature are commonly explained as due to magnetizing interactions or cubic anisotropy.^28^ From the macroscopic magnetic property analysis, it was impossible to determine the cause of the observed behavior of the samples. Here, using SANSPOL, we can follow the change in magnetized volume for each sample, giving access to the possible surface spin disorder contribution. The results from the SANSPOL nuclear interference term show that samples Co86Mn14, Co74Mn26, and Co37Mn63 exhibit a magnetically disordered layer on the surface. Combining these observations with the previous results obtained from Δ*M* plots (see our previous results^24^), we could conclude that the negative values of Δ*M* are due to the inhomogeneity of the spin structure at the surface.

From the *M*_*z*_(*H*) and magnetized volume, the disorder energy can be calculated according to eq 1 in the Supporting Information (SI).^5,23^ From the derivative of the disorder energy with respect to the magnetized volume (eq 2 in SI), we can obtain the *K*_eff–surf_, the surface contribution to the total effective anisotropy. Table 2 reports that *K*_eff–surf_ decreases with increasing Mn content in the ferrite structure. Moreover, we calculated the surface anisotropy constant according to Zákutná et al.^23^ (eq 3 in SI) and found that it is more significant for the Co-richest sample but stays constant for the other Co/Mn ratios.

**Table 2:** Obtained Values of Effective Anisotropy, *K*,^24^ Effective Surface Anisotropy, *K*_eff–surf_, Maximum Surface Anisotropy, *K*_s_, Maximum Dipole–Dipole Interaction Contribution, *K*_dd–max_, Shape Anisotropy Contribution, *K*_shape_, and Remaining Magnetocrystalline Anisotropy, *K*_A_

parameter	Co86Mn14	Co74Mn26	Co37Mn63	Co23Mn77
*K* (10^5^ J m^–3^)	10.9(4)	9.99(4)	6.37(3)	3.74(2)
*K*_eff–surf_ (10^5^ J m^–3^)	5.4(3)	4.0(5)	3.5(5)	3.5(9)
*K*_s_ (mJ m^–2^)	0.82(4)	0.60(7)	0.55(8)	0.6(2)
*K*_dd–max_ (10^5^ J m^–3^)	0.054	0.041	0.026	0.024
*K*_shape_ (10^5^ J m^–3^)	0.62(6)	0.14(3)	0.23(3)	0.21(2)
*K*_A_ (10^5^ J m^–3^)	4.8(5)	5.8(5)	2.7(5)	–0.01(1)

**Figure 4:**
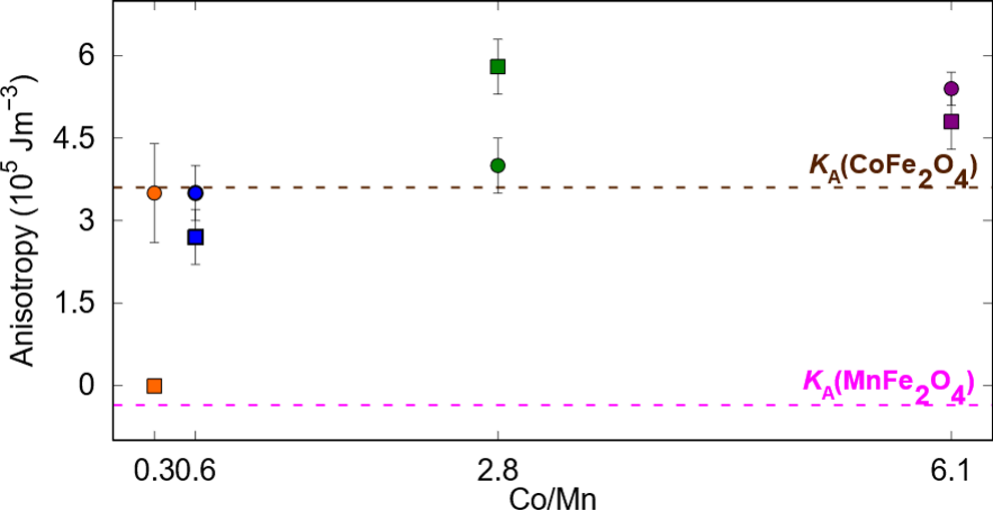
Trend of *K*_eff–surf_ (circles) and *K*_A_ (squares) as a function of the Co/Mn ratio. Pink and brown dashed lines indicate the bulk *K*_A_ values for MnFe_2_O_4_ and CoFe_2_O_4_, respectively.

Our previous study showed that surface anisotropy does not depend on the size of NPs;^5^ moreover, here, we have clear confirmation that the chemical composition can also alter the surface anisotropy contribution, as already demonstrated in previous studies.^29^ Knowing the effective anisotropy constant from averaged magnetization measurements^24^ and the averaged surface anisotropy contribution from SANSPOL allows us to extract the magnetocrystalline anisotropy constant and estimate the effect of Co/Mn content in the spinel structure. As shown in Figure 4, *K*_eff–surf_ increases with the Co/Mn ratio. Moreover, it is worth noting that the *K* is obtained from powder measurements where non-negligible interparticle interactions might be present in contrast to SANSPOL. In an earlier investigation of the magnetic interactions in Co-doped MnFe_2_O_4_, the dipolar interaction was observed in all of the samples under study.^29^ Hence, we calculated the maximum dipole–dipole interaction contributions and compared them with the different anisotropy contributions. The contribution of interparticle interactions to the *K* was estimated using the dipole–dipole interactions energy (*E*_dd_ = μ_0_μ^2^/(4π*r*_ptp_^3^)). Considering the particle-to-particle distance, *r*_ptp_, in the closest proximity when the NPs are separated by an OA ligand leads to values of interparticle anisotropy, *K*_dd-max_, of 10^3^ order of magnitude (see Table 2), which is negligible compared to surface anisotropy. An additional contribution to the total effective anisotropy arises from the shape of nanoparticles. Indeed, looking at HRSTEM micrographs (inset of Figure 2; for more information, see the SI), the morphology of NPs is not perfectly spherical, but the majority of NPs are faceted. Taking into account the cuboidal shape of NPs (having (220) facets at the surface), the shape anisotropy constant can be expressed as , where *N*_*zz*_ and *N*_*xx*_ are demagnetization tensor eigenvalues (for more information, see the SI).

As we can see from HRSTEM micrographs, none of the NPs have a perfect spherical morphology but rather a cuboidal shape with a mean aspect ratio in the range of 1.06–1.13 (see the SI). The obtained values of shape anisotropy (Table 2) were subtracted from the total effective anisotropy, leading to the derived values of magnetocrystalline anisotropies (*K*_A_). As shown in Figure 4, the *K*_A_ increases with the Co/Mn ratio. The reported room temperature bulk values of *K*_A_ from pure CoFe_2_O_4_ and MnFe_2_O_4_ are 3.6 × 10^5^ and −0.36 × 10^5^ J m^–3^, respectively.^30,31^ Looking at the obtained dependence of *K*_A_ with the Co/Mn ratio, we can see that two samples (Co23Mn77 and Co37Mn26) are in the expected range of magnetocrystalline anisotropy. This result aligns with previously published results, where the anisotropy constant is observed to decrease with the amount of manganese due to the replacement of Co by Mn in the octahedral sites.^24^ Co74Mn26 and Co86Mn14 samples have a high spinel inversion degree of 0.76 compared to the Co37Mn63 and Co23Mn77 samples, where the inversion degree is significantly smaller with a value of 0.57, which also affects the magnetocrystalline anisotropy. However, in the case of the Co74Mn26 and Co86Mn14 samples, we have additionally noticed a large number of defects and grain boundaries in the crystal structure (see the SI) that might further affect the magnetocrystalline anisotropy, which can lead to the enhancement of anisotropy associated with translational bond breaking, spin–orbit interaction, and enhancement of superexchange interaction.

## Conclusions

In conclusion, a series of mixed Co/Mn ferrites NPs having Co/Mn molar ratios of 6.1, 2.8, 0.6, and 0.3 and similar size and polydispersity were analyzed using SANSPOL to shed light on the behaviors observed from IRM-DCD protocols in our previous study. The samples with Co/Mn 0.6, 2.8, and 6.1 exhibited similar responses showing negative Δ*M* values, while the Mn-richest sample (Co/Mn of 0.3) showed positive values of Δ*M*. Using SANSPOL, we observed that the samples with negative Δ*M* values showed the presence of a magnetically disordered layer, while the Mn-richest sample did not show any increase in the magnetic radius with the applied magnetic field. Additionally, the study isolated different contributions to the total magnetic anisotropy, highlighting the significant role of shape anisotropy due to the faceted morphology of the NPs. These findings enhance our understanding of the complex interplay between magnetic interactions, surface structure, and anisotropy in mixed Co/Mn ferrites, offering more profound insights into tuning their magnetic properties, which are important for AC biomedical applications, such as cancer treatment by magnetic fluid hyperthermia.

## Supporting Information Available

Magnetization results, instrumentation part, SANSPOL *I*_Q_^–^ and *I*_Q_^+^ scattering cross sections with the core–shell-surfactant model fit and obtained radial distribution of magnetic scattering length density with summarized results from SANSPOL *I*_Q_^–^ and *I*_Q_^+^ scattering cross sections and nuclear-magnetic interference term fits. HRSTEM and conventional TEM micrographs with the estimation of the aspect ratio, and description of the calculation of disorder energy, surface, and shape anisotropy constant (PDF)
