# Supplementary material for: Exploring Anisotropy Contributions in MnxCo1–xFe2O4 Ferrite Nanoparticles for Biomedical Applications
Source: ACS Appl Nano Mater. 2024 Nov 15;7(23):27210–6. doi: 10.1021/acsanm.4c05231 (PMC11650618; doi:10.1021/acsanm.4c05231)
Supplement: Supplementary file 1 — an4c05231_si_001.pdf [file an4c05231_si_001.pdf]

Exploring Anisotropy Contributions in $Mn_xCo_{1-x}Fe_2O_4$ Ferrite Nanoparticles for Biomedical Applications

Marianna Gerina[1], Marco Sanna Angotzi[2], Valentina Mameli[2], Michal Mazur[3], Nicoletta Rusta[2], Elena Balica[4], Pavol Hrubovcak[5], Carla Cannas[2], Dirk Honecker[6], and Dominika Zákutná[1]

[1]Department of Inorganic Chemistry, Charles University, Hlavova 8, 12800 Prague 2, Czech Republic
[2]Department of Chemical and Geological Sciences, University of Cagliari, S.S. 554 bivio per Sestu, 09042 8 Monserrato (CA), Italy
[3]Department of Physical and Macromolecular Chemistry, Charles University, Hlavova 2030/8, 128 43 Prague 2, Czech Republic
[4]Dipartimento di Chimica and INSTM, Universita'di Firenze, Via della Lastruccia 3, I- 50019 Sesto Fiorentino, Italy
[5]Institute of Physics, Faculty of Science, P.J. Šafárik University, Park Angelinum 9, 04001 Košice, Slovakia
[6]ISIS Neutron and Muon Facility, Science and Technology Facilities Council,Rutherford Appleton Laboratory, OX11 0QX Didcot, United Kingdom
E-mail: zakutnad@natur.cuni.cz

November 14, 2024

Preliminary Results

In **Table S1**, selected results are summarized from our previous studies for better comparison[1].

Table S 1: Summarized results from our previous study[1]. T_{max}, T_{diff}, T_{B}, $\mu_0 H_{\mathrm{C}}$, and $M_{\mathrm{S}}^{300\mathrm{K}}$ represent the maximum, furcation point of the ZFC curve (2% of difference), blocking temperature, coercive field at 10 K, and saturation magnetization at the 300 K, respectively.

**Parameter** / **Sample**	T_{max} (K)	T_{diff} (K)	T_{B} (K)	$\mu_0 H_{\mathrm{C}}$ (T)	$M_{\mathrm{S}}^{300\mathrm{K}}$ ($\mathrm{Am^2kg^{-1}}$)
**Co23Mn77**	231(5)	235(5)	171(3)	0.65(1)	74(2)
**Co37Mn63**	259(5)	280(6)	194(4)	1.12(3)	77(2)
**Co74Mn26**	282(6)	314(6)	197(4)	1.76(1)	79(2)
**Co86Mn14**	315(6)	328(7)	234(5)	1.92(1)	80(2)

Experimental

SAXS

Small-angle X-ray scattering (SAXS) experiments were executed at the Department of Mathematics and Physics of the Charles University in Prague using a Xenocs Xeus 2.0 equipped with Cu and Mo K_α microfocus X-ray sources, toroidal parallel beam producing X-ray mirrors, two sets of beam collimating scatter-less slits and Dectris Pilatus 200k detector with detector distance of 2.50 m using both Cu and Mo wavelengths to cover the maximum accessible Q-range. Measured 2D intensity profiles were azimuthally integrated. We corrected the 1D SAXS patterns to the capillary/sample thickness and the transmission of samples, and we subtracted solvent and capillary signal. The samples were analyzed as toluene dispersion with a concentration of 3.5 mg/mL in a borosilicate glass capillary with 1.5 mm diameter, 80 mm length, and 0.01 mm wall thickness.

SANS

SANS experiments (RB2220620-1)[2] were performed at the LARMOR instrument (ISIS, UK) with a horizontally applied magnetic field (perpendicular to the neutron beam) in the range of 9.5 mT - 1.5 T (saturation field of 1.5 T). The neutron beam was polarized by a supermirror polarizer, and incident beam polarization (efficiency of better than 0.95 for neutrons above 2.8 Å) was reversed by a radio-frequency flipper (efficiency of 0.99). The samples were analyzed as dispersions in d8-toluene in a quartz disc-shaped "banjo" cell. The nuclear scattering cross-section was obtained from the 20° sector integration along the applied magnetic field direction at the saturation field of the NPs. The scattering cross-sections with different incident neutron beam polarization were fully radially averaged for analysis. The nuclear-magnetic interference term was obtained as difference of $I_{\mathrm{Q}}^{+} - I_{\mathrm{Q}}^{-}$ 2 D scattering cross-sections followed by full radial averaging. The data reduction was done in Mantid software[3], and the data analysis was performed in a GUI interface available on GitHub[4].

HRSTEM

High-resolution scanning TEM (HRSTEM) measurements were carried out on JEOL NEOARM 200 F operating at 200 kV equipped with Schottky-FEG cathode and CS corrector, respectively. The toluene dispersion of NPs was dropped at the Cu grid with 400 mesh coated by carbon foil. Acquisition of HRSTEM micrographs was made in annular bright (ABF) and dark-field (ADF) mode.

Results from magnetic SANS

SANSPOL I_Q^- and I_Q^+ scattering cross-sections with the core-shell-surfactant model fit and obtained radial distribution of magnetic scattering length density is presented in **Figure S1** with summarized results in **Table S2**.

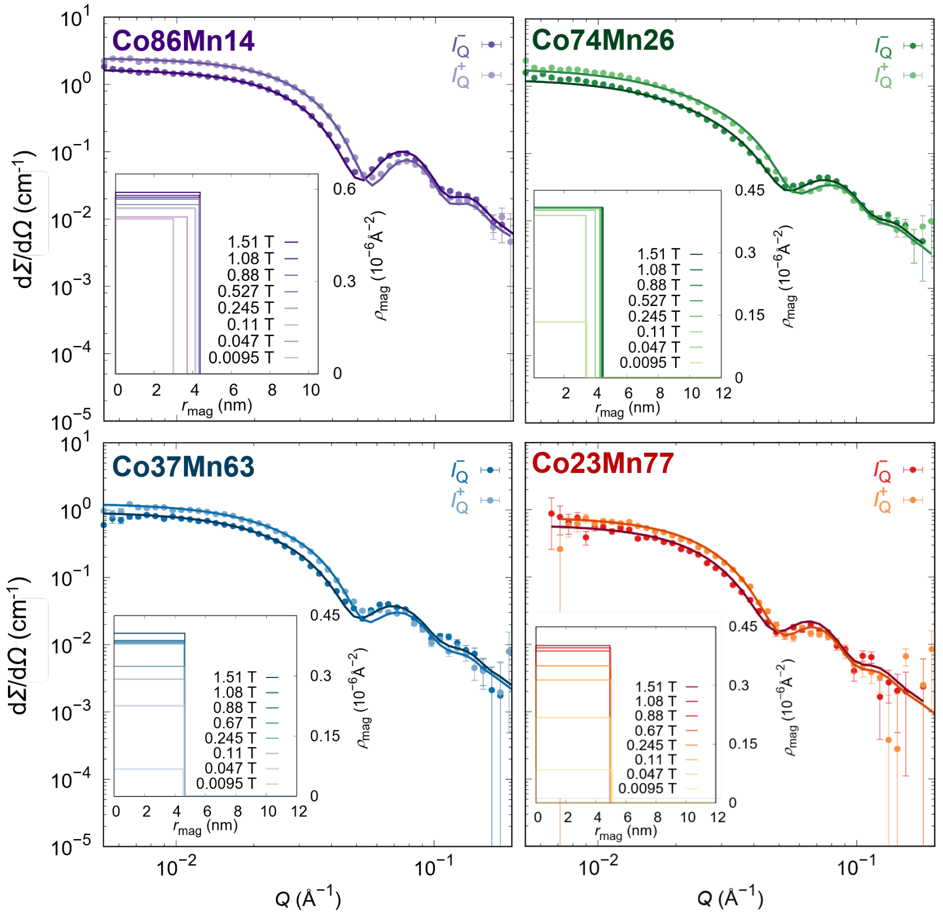

Figure S 1: SANSPOL scattering cross sections (points) for the polarization I_Q^- and I_Q^+ with core-shell-dead layer form factor refinements (full lines) at 1.51 T. Insets: Obtained radial distribution of ρ_{mag} at different applied magnetic fields.

Table S 2: Summarized results from SANSPOL refinements at different applied magnetic fields, B, where r_{mag}, d_{dis}, ρ_{mag}, M_{z}, M_{ave}, ΔE_{dis} and $K_{\mathrm{eff-surf}}$ correspond to the magnetic radius, disorder thickness, magnetic scattering length density of core, longitudinal magnetization, averaged magnetization, disorder energy and surface contribution to K_{eff}, respectively.

Parameter / Sample	B (T)	r_{mag} (nm)	d_{dis} (Å)	ρ_{mag} (10^{-7} Å^{-2})	M_{z} (kA/m)	M_{ave} (kA/m)	ΔE_{dis} (10^{-20} J)	$K_{\mathrm{eff-surf}}$ (10^{5} Jm^{-3})
**Co86Mn14**	1.51	4.35(2)	3.5(2)	5.91(7)	402(5)	322(6)	-0.44(5)	6.1(9)
	1.08	4.37(2)	3.3(2)	5.64(6)	388(4)	388(7)	-0.10(1)	4.2(6)
	0.86	4.38(2)	3.2(2)	5.59(5)	384(4)	310(5)	-	-
	0.53	4.39(2)	3.1(2)	5.46(5)	376(4)	306(5)	0.048(5)	2.0(3)
	0.25	4.40(2)	3.0(2)	5.18(7)	356(5)	292(6)	0.051(5)	0.9(1)
	0.11	4.40(4)	3.0(6)	4.58(9)	315(6)	258(8)	0.020(2)	0.36(6)
	0.047	4.39(6)	3.0(6)	3.4(1)	232(8)	189(11)	0.0042(5)	0.11(2)
	0.0095	4.4(2)	3(2)	2.1(2)	142(12)	115(15)	-	-
**Co74Mn26**	1.51	4.49(2)	0.068(5)	4.1(1)	280(8)	276(8)	8.7(2)	4.2(1)
	1.08	4.46(4)	0.41(3)	4.1(1)	281(8)	274(7)	6.0(2)	3.04(8)
	0.86	4.41(4)	0.88(3)	4.1(1)	279(7)	263(7)	4.5(1)	2.46(7)
	0.53	4.36(4)	1.41(2)	4.1(1)	280(7)	255(6)	2.55(7)	1.48(4)
	0.25	4.27(4)	2.26(3)	4.0(1)	279(7)	239(6)	1.04(3)	0.68(2)
	0.11	3.99(4)	5.10(3)	4.0(1)	276(7)	193(5)	0.29(7)	0.32(8)
	0.047	3.41(4)	10.994(3)	3.9(1)	268(7)	116(3)	0.011(3)	-0.0.13(3)
	0.0095	3.47(4)	10.35(4)	1.4(1)	93(8)	43(4)	-	-
**Co37Mn63**	1.51	4.64(5)	3.6(5)	4.1(1)	279(7)	224(9)	1.1(3)	4(2)
	1.08	4.63(5)	3.7(5)	3.9(1)	267(7)	212(9)	0.6(2)	3(1)
	0.86	4.61(5)	3.9(5)	3.8(1)	264(7)	206(9)	0.4(1)	2.3(9)
	0.66	4.63(5)	3.7(5)	3.8(1)	262(7)	207(9)	0.4(1)	1.8(6)
	0.25	4.62(6)	3.8(6)	3.3(1)	223(7)	176(9)	0.10(3)	0.5(2)
	0.11	4.58(7)	4.2(7)	2.9()	201(7)	155(10)	0.02(6)	0.2(8)
	0.047	4.59(9)	4.1(9)	2.3(1)	156(7)	121(9)	0.009(2)	0.07(3)
	0.0095	4.55(4)	5(4)	0.7(1)	47(9)	35(16)	-	-
**Co23Mn77**	1.51	5.20(9)	2.0(9)	3.5(1)	241(10)	216(15)	0.18(4)	3(1)
	1.08	5.18(7)	2.2(7)	3.6(1)	248(8)	220(12)	-0.036(8)	2.7(8)
	0.86	5.18(8)	2.2(8)	3.6(1)	249(9)	220(13)	-0.059(1)	2.2(7)
	0.66	5.16(9)	2.4(9)	3.6(1)	247(10)	216(14)	-0.14(3)	1.7(5)
	0.25	5.18(9)	2.2(9)	3.3(1)	225(9)	199(13)	-0.015(3)	0.6(2)
	0.11	5.1(1)	3(1)	3.0(1)	204(9)	176(13)	-0.037(8)	0.23(7)
	0.047	5.1(1)	3(1)	2.1(1)	142(9)	119(12)	-0.022(5)	0.07(5)
	0.0095	5.2(4)	2(4)	0.8(1)	56(10)	50(14)	-	-

Results from the nuclear-magnetic interference term refined with the core-shell-surfactant model are summarized in **Table S3**.

Table S 3: Summarized results from nuclear-magnetic interference term at different applied magnetic fields, B, where $r_{\rm mag}$, $d_{\rm dis}$, $\rho_{\rm mag}$, $M_{\rm z}$, $M_{\rm ave}$, $\Delta E_{\rm dis}$ and $K_{\rm eff-surf}$ correspond to the magnetic radius, disorder thickness, magnetic scattering length density of core, longitudinal magnetization, averaged magnetization, disorder energy and surface contribution to $K_{\rm eff}$, respectively.

**Parameter** / **Sample**	B (T)	$r_{\rm mag}$ (nm)	$d_{\rm dis}$ (Å)	$\rho_{\rm mag}$ (10^{-7} Å^{-2})	$M_{\rm z}$ (kA/m)	$M_{\rm ave}$ (kA/m)	$\Delta E_{\rm dis}$ (10^{-20} J)	$K_{\rm eff-surf}$ (10^5 Jm^{-3})
**Co86Mn14**	1.51	4.50(1)	2.0(1)	5.27(3)	362(2)	318(3)	12.6(5)	5.5(3)
	1.08	4.62(1)	0.8(1)	4.75(3)	326(2)	310(3)	9.3(4)	3.5(2)
	0.86	4.62(1)	0.8(1)	4.74(3)	326(2)	309(3)	7.5(3)	2.9(2)
	0.53	4.61(1)	0.9(1)	4.66(3)	320(2)	302(3)	4.4(2)	1.7(9)
	0.25	4.59(1)	1.1(1)	4.54(3)	312(2)	290(4)	1.94(7)	0.76(4)
	0.11	4.52(2)	1.82(62)	4.18(3)	287(2)	255(3)	0.78(3)	0.33(2)
	0.047	4.04(2)	6.6(2)	4.12(6)	283(4)	179(4)	0.166(7)	0.13(8)
	0.0095	3.30(4)	14.0(4)	4.0(1)	275(8)	95(5)	-	-
**Co74Mn26**	1.51	4.49(3)	0.1(3)	3.84(8)	264(6)	262(8)	11(1)	4.0(5)
	1.08	4.49(3)	0.1(3)	3.77(8)	259(6)	257(8)	7.9(7)	2.8(3)
	0.86	4.45(4)	0.5(5)	3.74(1)	257(5)	249(8)	6.20(6)	2.3(3)
	0.53	4.40(4)	1.0(4)	3.74(8)	257(5)	241(8)	3.5(3)	1.4(2)
	0.25	4.32(4)	1.9(4)	3.72(9)	256(6)	224(8)	1.5(1)	0.6(8)
	0.11	4.02(4)	4.8(4)	3.72(8)	256(5)	182(6)	0.52(4)	0.29(3)
	0.047	3.43(5)	10.7(5)	3.6(1)	249(8)	110(6)	0.085(8)	0.12(2)
	0.0095	2.84(8)	16.6(8)	1.9(1)	131(9)	33(4)	-	-
**Co37Mn63**	1.51	4.69(2)	3.15(2)	3.38(4)	232(3)	191(4)	14(1)	3.5(5)
	1.08	4.60(2)	4.0(2)	3.38(4)	232(3)	181(4)	9(1)	2.5(4)
	0.86	4.56(3)	4.4(3)	3.37(5)	232(3)	176(4)	7.2(2)	2.0(3)
	0.66	4.57(2)	4.3(2)	3.33(4)	229(3)	175(3)	5.5(6)	1.5(2)
	0.25	4.29(3)	7.1(3)	3.31(5)	227(3)	144(3)	1.6(2)	0.56(9)
	0.11	4.14(3)	8.6(3)	3.22(6)	221(4)	126(4)	0.64(7)	0.26(4)
	0.047	3.71(4)	12.9(4)	3.16(8)	217(5)	86(4)	0.17(1)	0.10(2)
	0.0095	2.19(8)	28.1(8)	3.0(2)	209(2)	18(2)	-	-
**Co23Mn77**	1.51	5.25(6)	1.5(6)	3.37(9)	232(6)	213(10)	18(5)	3.5(9)
	1.08	5.25(6)	1.5(6)	3.43(9)	236(6)	217(10)	13(2)	2.5(6)
	0.86	5.26(8)	1.4(8)	3.4(1)	234(7)	216(11)	11(2)	2.1(5)
	0.66	5.25(6)	1.5(6)	3.41(9)	234(6)	216(9)	8(1)	1.6(4)
	0.25	5.04(6)	3.6(6)	3.36(1)	231(7)	187(7)	2.6(5)	0.57(1)
	0.11	4.82(5)	5.8(5)	3.36(9)	231(6)	164(7)	1.1(2)	0.27(7)
	0.047	4.17(8)	12.3(8)	3.3(2)	227(1)	105(8)	0.3(5)	0.11(3)
	0.0095	2.6(1)	28(1)	3.0(4)	204(3)	23(5)	-	-

Disorder energy and surface anisotropy constant

The disorder energy, E_{dis} is defined as

$$E_{\mathrm{dis}} = \mu \cdot H \cdot M_{\mathrm{z}}(H) \cdot [V_{\mathrm{mag}}(H) - V_{\mathrm{mag}}(H_{\mathrm{min}})], \tag{1}$$

where $M_{\mathrm{z}}(H)$, $V_{\mathrm{mag}}(H_{\mathrm{max}})$ and $V_{\mathrm{mag}}(H_{\mathrm{min}})$ are the longitudinal magnetization at the applied field, and magnetized volumes at H_{max} and H_{min}, respectively. Afterward, they accessed the surface contribution to effective anisotropy constant according to the following equation:

$$K_{\mathrm{eff-surf}} = \frac{\partial E_{\mathrm{dis}}}{\partial V_{\mathrm{mag}}}, \tag{2}$$

where $\partial E_{\mathrm{dis}}$ and $\partial V_{\mathrm{mag}}$ are the derivative of the disorder energy and of the magnetic volume, respectively. From the effective anisotropy constant, the spatially resolved surface anisotropy constant, K_{S}, can be obtained:

$$K_{\mathrm{S}} = K_{\mathrm{eff-surf}} \cdot \frac{r_{\mathrm{mag}}}{3} \tag{3}$$

HRSTEM micrographs

HRSTEM micrographs of all samples in ADF mode are presented in **Figure S2** and histogram of aspect ratio with lognormal distribution function in **Figure S3**.

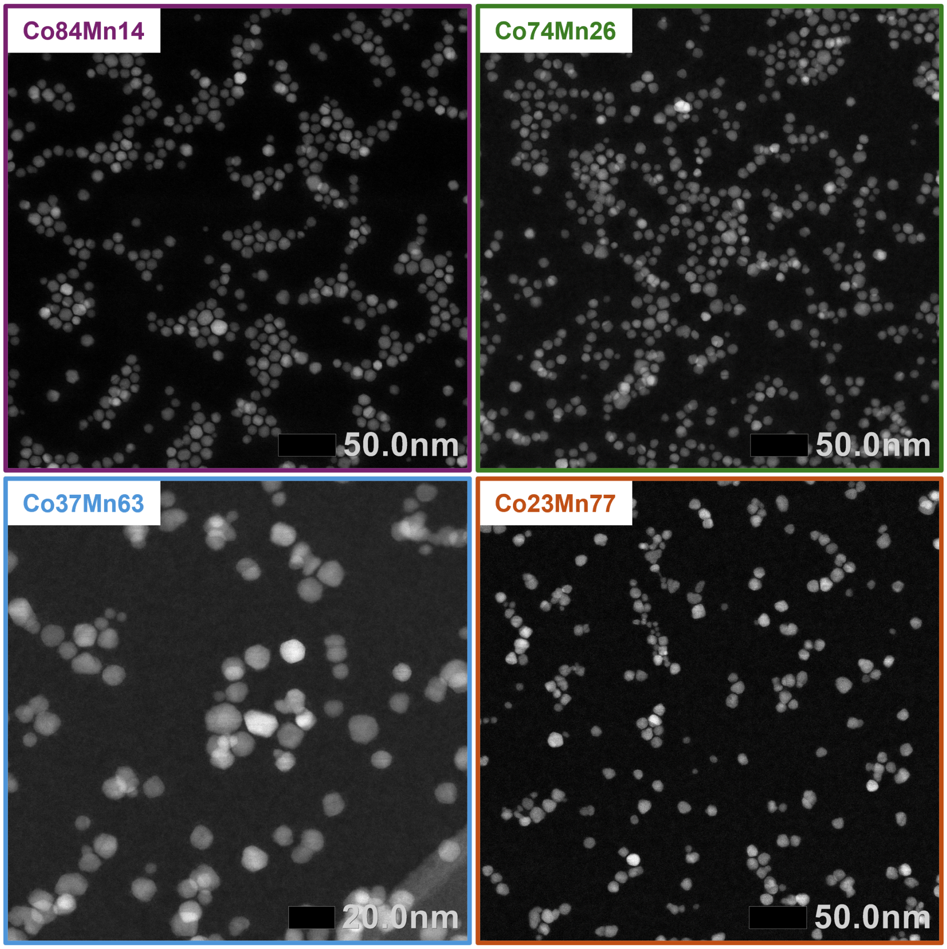

Figure S 2: HRSTEM micrographs in ADF mode for the samples Co86Mn14, Co74Mn26, Co37Mn63, and Co23Mn77.

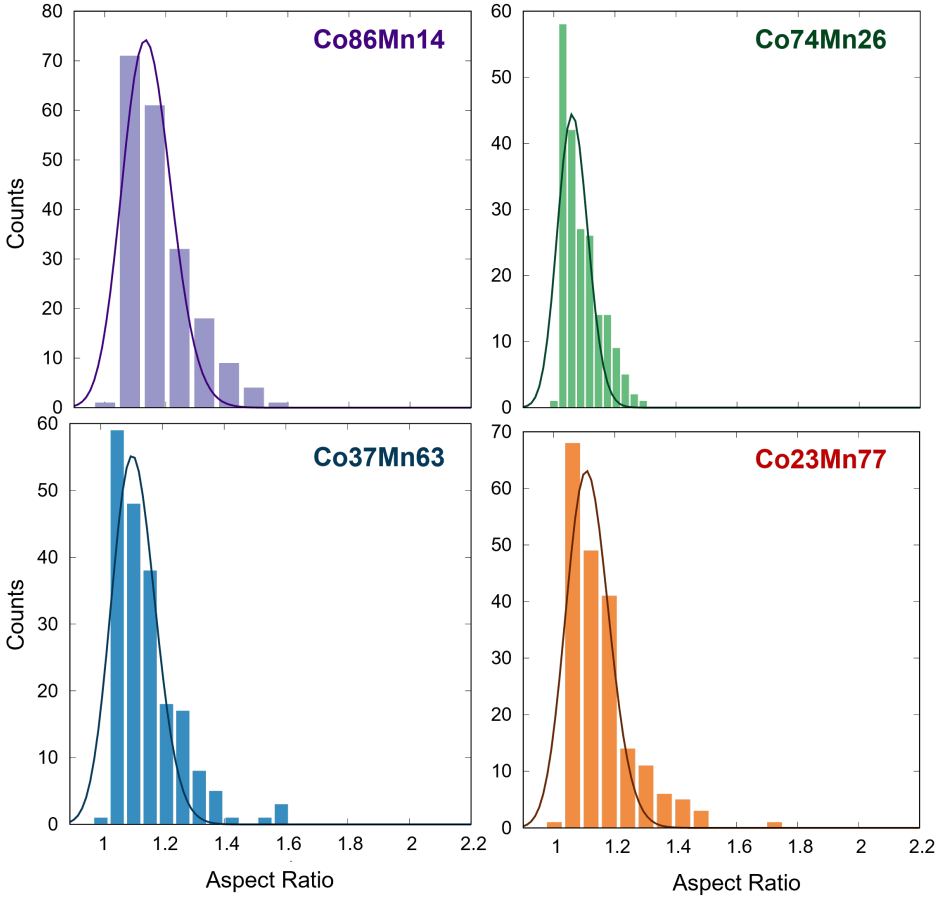

Figure S 3: Distribution of aspect ratio of the nanoparticles obtained from TEM micrograph analysis with lognormal distribution function fit.

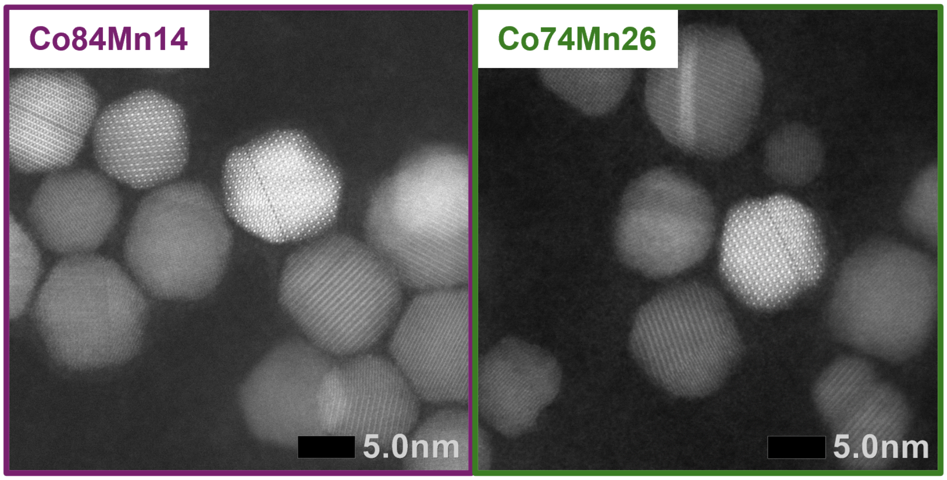

Figure S 4: Detail on defects in the crystal structure of Co86Mn14 and Co74Mn26 samples.

Shape anisotropy

The anisotropic shape of particles affects the demagnetizing energy accordingly:

$$E_{\mathrm{d}} = -\frac{1}{2}\mu_0 M H_{\mathrm{d}} \tag{4}$$

with $\mu_0 = 4\pi \cdot 10^{-7}\,\mathrm{H/m}$ as a permeability of free space, H_{d} is a demagnetization field, which depends on the elongation with a symmetry trace:

$$E_{\mathrm{d}} = -\frac{\mu_0}{2}\begin{pmatrix} M_{\mathrm{x}} & M_{\mathrm{y}} & M_{\mathrm{z}} \end{pmatrix}\begin{pmatrix} N_{\mathrm{xx}} & 0 & 0 \\ 0 & N_{\mathrm{yy}} & 0 \\ 0 & 0 & N_{\mathrm{zz}} \end{pmatrix}\begin{pmatrix} M_{\mathrm{x}} \\ M_{\mathrm{y}} \\ M_{\mathrm{z}} \end{pmatrix} \tag{5}$$

that leads to:

$$E_{\mathrm{d}} = -\frac{\mu_0}{2}(N_{\mathrm{xx}}M_{\mathrm{x}}^2 + N_{\mathrm{yy}}M_{\mathrm{y}}^2 + N_{\mathrm{zz}}M_{\mathrm{z}}^2) \tag{6}$$

for uniformly magnetized nanoparticles, the perpendicular components of the demagnetizing energy are the same. If we consider the change in energy due to magnetization rotation in the x-z plane, the z and x components of the magnetization are related by $M_{\mathrm{x}}^2 = (1 - M_{\mathrm{z}})^2$ and by applying $M_{\mathrm{z}} = m_{\mathrm{z}}M_{\mathrm{S}}$ the demagnetizing energy has a form of shape anisotropy:

$$K_{\mathrm{shape}} = -\frac{\mu_0 M_{\mathrm{S}}}{2}(N_{\mathrm{xx}} - N_{\mathrm{zz}})m_{\mathrm{z}}^2 \tag{7}$$

where for prolate spheroid the N_{xx} and N_{zz} are given[5, 6]:

$$N_{\mathrm{zz}} = \frac{4\pi}{A^2 - 1}(\frac{A}{\sqrt{A^2 - 1}}\ln(A + \sqrt{A^2 - 1}) - 1) \tag{8}$$

$$N_{\mathrm{xx}} = \frac{4\pi - N_{\mathrm{zz}}}{2} \tag{9}$$

where A is the aspect ratio.

References

(1) Sanna Angotzi, M.; Mameli, V.; Zákutná, D.; Kubániová, D.; Cara, C.; Cannas, C. *J. Phys. Chem. C* **2021**, *125*, 20626–20638.

(2) Mameli, V.; Sanna Angotzi, M.; Balica, E.; Hrubovčák, P.; Zákutná, D.; Rusta, N.; Smith, G.; Gerina, M. 14 November 2022, Unravelling the chemical dependence of spin disorder phenomena and interactions in Co-Mn mixed spinel ferrites nanoparticles, *STFC ISIS Neutron and Muon Source* `https://doi.org/10.5286/ISIS.E.RB2220620-1`.

(3) Arnold, O. et al. *Nucl. Instrum. Methods Phys. Res. A.* **2014**, *764*, 156–166.

(4) Dresen, D. `https://github.com/DomiDre/modelexp`.

(5) Vallejo-Fernandez, G.; O'Grady, K. *Appl. Phys. Lett.* **2013**, *103*, 142417.

(6) Moreno R. Poyser, S.; Meilak, D. *Sci. Rep.* **2020**, *10*, 2722.
